# A machine learning model identifies M3-like subtype in AML based on PML/RARα targets

**DOI:** 10.1016/j.isci.2024.108947

**Published:** 2024-01-18

**Authors:** Tingting Shao, Jianing Li, Minghai Su, Changbo Yang, Yingying Ma, Chongwen Lv, Wei Wang, Yunjin Xie, Gang Xu, Ce Shi, Xinying Zhou, Huitao Fan, Yongsheng Li, Juan Xu

**Affiliations:** 1 College of Bioinformatics Science and Technology, Harbin Medical University, Harbin, Heilongjiang Province 150001, China; 2 School of Interdisciplinary Medicine and Engineering, Harbin Medical University, Harbin 150001, China; 3 Key Laboratory of Hepatosplenic Surgery of Ministry of Education, NHC Key Laboratory of Cell Transplantation, the First Affiliated Hospital, Harbin Medical University, Harbin, Heilongjiang Province 150001, China; 4 The Second Affiliated Hospital, Harbin Medical University, Harbin, Heilongjiang Province 150001, China

**Keywords:** Medical science, Genomics, Machine learning

## Abstract

The typical genomic feature of acute myeloid leukemia (AML) M3 subtype is the fusion event of PML/RARα, and ATRA/ATO-based combination therapy is current standard treatment regimen for M3 subtype. Here, a machine-learning model based on expressions of PML/RARα targets was developed to identify M3 patients by analyzing 1228 AML patients. Our model exhibited high accuracy. To enable more non-M3 AML patients to potentially benefit from ATRA/ATO therapy, M3-like patients were further identified. We found that M3-like patients had strong GMP features, including the expression patterns of M3 subtype marker genes, the proportion of myeloid progenitor cells, and deconvolution of AML constituent cell populations. M3-like patients exhibited distinct genomic features, low immune activity and better clinical survival. The initiative identification of patients similar to M3 subtype may help to identify more patients that would benefit from ATO/ATRA treatment and deepen our understanding of the molecular mechanism of AML pathogenesis.

## Highlights

• A method based on PML/RARα targets was developed to identify M3 patients
• Computational model helps identifying M3 patients with AUCs ranged from 0.965 to 1.00
• M3-like patients were with GMP features and would benefit from ATO/ATRA treatment

## Graphical abstract

**Figure:**
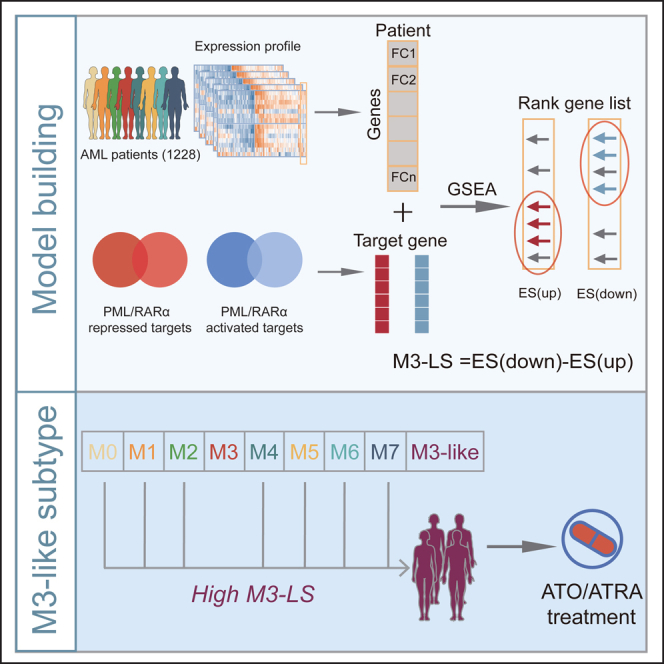

## Introduction

Acute myeloid leukemia (AML) results from the clonal expansion of hematopoietic precursor cells with disease-causing genetic mutations or chromosomal changes. Previous studies have characterized distinct molecular subpopulations of AML, and the presence of certain disease-associated mutations are currently used to classify the disease, predict prognosis, and in some cases guide treatment.^1^^,^^2^^,^^3^ Acute promyelocytic leukemia (APL) is a distinct subtype of AML characterized by the expansion and accumulation of leukemic cells that are blocked at the promyelocytic stage of granulocyte differentiation, as well as the presence of a specific disease driver fusion gene encoding the PML/RARα oncoprotein.^4^ An atlas of PML/RARα direct targets has been identified, which redefined the activating function that acted through super-enhancers and explained synergism of ATRA/ATO.^5^ Morphologically, APL is recognized as M3 subtype of AML by the French-American-British classification. Over the past two decades, all-trans retinoic acid (ATRA) and arsenic trioxide (ATO)-based combination (ATRA-ATO) treatment has been shown to be more effective and have become the standard core treatment regimen for APL today.^6^^,^^7^^,^^8^ Among various subtypes of AML, M3 subtype has the highest survival rate,^9^ which is attributed to the combination therapy of ATRA and ATO. ATRA and ATO trigger degradation of PML/RARα, thereby inhibiting disease progression, while non-M3 AMLs have a mixed response to this combination therapy.^10^^,^^11^ We would like to explore which non-M3 AML patients may benefit from ATRA and ATO combination therapy through in-depth study of PML/RARα target genes.

In the genetics of myeloid tumors, chromosomal translocations usually involve transcription factors (TFs), which lead to abnormal regulation of downstream target genes by oncogenic fusion TFs, induce malignant cells proliferation, and interfere with bone marrow differentiation.^12^ As the most important oncogenic fusion in APL, both *PML* and *RARα* are TFs, and can directly *trans*-activate some essential oncogenes, which play important roles in disease progression of APL.^13^^,^^14^ On the other hand, PML/RARα can also suppress the expression of some tumor suppressor genes. For example, PML/RARα inhibits PU.1-dependent activation of immune subunits, thereby contributing to the escape of APL cells from immune surveillance.^15^^,^^16^ Both ATRA and ATO directly target PML/RARα-mediated transcriptional repression and protein stability. ATRA targets the RARα part of the fusion, while ATO targets the PML part,^17^^,^^18^ which promotes the degradation of PML/RARα and then affects the expression of its downstream target genes, such as *WT1*, *C/EBPbeta*, and *GATA2*.^19^^,^^20^^,^^21^ These genes are important for APL cell differentiation or proliferation. In M3 subtype patients, the combination therapy approach has a synergistic effect on the induction of myeloid differentiation and apoptosis. Moreover, in some non-M3 AML cell lines, ATRA-ATO combination therapy also has significant effects on leukemia cell differentiation induction and apoptosis.^22^^,^^23^^,^^24^^,^^25^ Overall, the successful usage of the ATRA-ATO combination therapy has good therapeutic efficacy and low drug resistance for M3 subtype patients and certain non-M3 AML cell lines. These results suggested that besides M3 subtype, other AML patients with similar expression patterns as M3 subtype ones might also benefit from the ATRA-ATO combination treatment strategy. The good therapeutic efficacy of ATRA-ATO might be not only dependent on the fusion event of PML/RARα, but also closely correlated with the expression features of its target genes.

Besides the PML/RARα fusion event, the expression or genomic patterns of several genes also aid to subtype characterization and treatment of AML. For example, the gene *FLT3*, which is mutated in approximately 40% of human APL cases, cooperates with PML/RARα in the development of the APL phenotype in mouse.^26^ As another example, the expression of peptidyl-Prolyl *cis*-trans isomerase *Pin1* is significantly increased in patients with various AMLs, including M3 subtype, which is discovered to be involved in a variety of cancer pathways in AML.^27^ Given these gene mutations and expression alteration, we believed that in addition to PML/RARα fusion events, other molecular alterations might also be involved in occurrence and progression of APL or AML. At present, the transcriptome characteristics of APL need to be further studied. Therefore, we believe that gene expression profiles can be used to explore similarities between M3 subtype and other AML subtypes, and those AML subtypes that are similar in gene expression to M3 subtype might deserve the same treatment strategy.

In this study, we found that PML/RARα targets tend to be differentially expressed in multiple AML subtypes and contribute to the classification of M3 subtype. Because the expression of PML/RARα targets is the downstream consequence of PML/RARα regulatory mechanisms, we hypothesized that our computational approach may aid to the current classification of M3 subtype from the view of transcriptome and further discover additional subpopulation therapeutic loopholes, which in turn could help identify pathogenic mechanisms. Therefore, an enrichment-based scoring index, defined as M3-Like Score (M3-LS), was developed to assess the expression pattern similarity of PML/RARα target genes in patients from non-M3 AML populations as M3 subtype. We further developed a classifier for identifying patients similar to M3 subtype with scores above a threshold according to Receiver operating characteristic curve (ROC) analysis. Moreover, by further requiring PML/RARα targets responded to ATRA/ATO or differentially expressed in M3 subtype, the performance of our classifier was improved. The robustness of our model was further validated in other independent AML populations. Notably, expression patterns of several vital marker genes in M3-like patients were discovered to be more concordant with M3 subtype. Moreover, we found that M3-like patients exhibited several features distinct from other non-M3 ones, including genomic mutation, molecular immune features, as well as survival prognosis. All these results indicated the need for identification of M3-like subtype based on transcriptome analysis, suggesting that these samples may also benefit from ATRA/ATO therapy.

## Results

### PML/RARα targets are perturbed across AMLs and help identify M3 subtype

**Figure 1:**
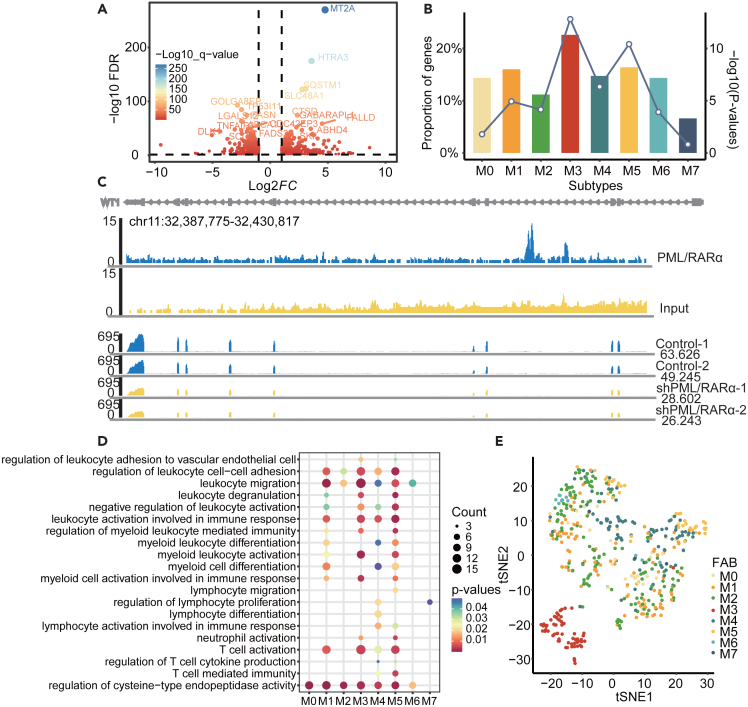
PML/RARα targets are perturbed across AMLs and help identify M3 subtype (A) The change of gene expression after PML/RARα gene knockout. The labels show the top 10 genes that are significantly upregulated or downregulated. (B) Enrichment of PML/RARα target genes and differential genes between AML patients and healthy control samples (FDR <0.05, FC > 1.5). The height of the bar graph is the proportion of differentially expressed genes in the targets, and the line chart is the -log10(p-value) of the hypergeometric test between the differential genes and the target genes. (C) Top: PML/RARα effects on transcriptional activities of the directly activated gene WT1. This diagram illustrates that ChIP-seq abundance in WT1 after PML/RARα knockout using small interfering RNA (siRNA) targeting the fusion site of PML/RARα. The panel shows the genome browser tracks of PML/RARα binding. Bottom: The abundance of RNA-seq was compared between two control samples and two samples using siRNA knockout of PML/RARα. The chip seq data were obtained from a previous study.^5^ (D) Functional enrichment analysis of differentially expressed PML/RARα target genes in each AML subtype. E. t-SNE analysis of PML/RARα target genes transcriptomic data for 519 AML samples in the training cohort. Each point represents a sample visualized in a two-dimensional projection. Samples of each subtype are displayed using a different color. Particularly, M3 subtype samples represented by red dots are spontaneously clustered together.

We respectively obtained 363 and 424 PML/RARα target genes that were significantly repressed and activated in M3 subtype from a previous study^5^ by integrating the transcriptome and regulation of PML/RARα in NB4 cell line (Figure 1A). Moreover, differential expression analysis was performed by comparing the expression of patients in different subtypes with normal samples (FDR <0.05, |FC| > 1.5) in the training AML cohort. We next evaluated whether PML/RARα target genes are likely to be enriched in these differentially expressed genes based on hypergeometric tests. As a result, we found that PML/RARα target genes were significantly enriched in M3 subtype (p < 1.55e-13), and approximately ∼22.62% targets were significantly abnormally expressed (Figure 1B). Notably, we found that the enriched p value in M3 subtype was most significant, and the significant enrichments were also observed in other subtypes (Figure 1B). When changing the thresholds of differential expression analysis, we obtained similar results (Figures S1A and S1B). Taking the target gene *WT1* as an example, Figure 1C showed the effect of PML/RARα on directly activated gene *WT1* (Figure 1C). It was significantly over-expressed in both M3 (FDR <2.84e-62, FC = 6.73) and several other subtypes (Figure S1C), which is known as a significant predictor of AML recurrence^28^ as well as an important marker for detection of AML minimal residues.^29^ We also found that the frequency of *WT1* mutation in the validation cohort-1 was 6.8%, and it was 4.5% in the M3 subtype. Only 0.25% of the target genes were differentially expressed between *WT1* mutation group and the wild-type group (Figure S2). These results suggest that *WT1* mutation has no significant effects on the expression of PML/RARα targets. *STAB1* as another example was also significantly up-regulated in M3 subtype (FDR <7.84e-89, FC = 5.46, Figure S1D), and reducing expression inhibits the growth of NB4 leukemia cells.^30^ *STAB1* was also a poor prognostic factor in AML, and the oncogenic functions have been confirmed in melanoma.^31^

To further understand the functional roles of PML/RARα target genes, we next performed functional enrichment analysis (FDR <0.05). We found that these differentially expressed targets across AML subtypes were significantly enriched in myeloid cell differentiation, activation, and immune regulation-related functions (Figure 1D). For example, differentially expressed PML/RARα target genes in M3 subtype were significantly enriched in leukocyte migration, myeloid leukocyte activation and T cell activation (Figure 1D). We further explored whether the expression patterns of PML/RARα targets can help distinguish patients in M3 subtype from other subtypes. We performed the tSNE dimensionality reduction and found that almost all patients in M3 subtype were not only clustered together, but also obviously distinguished from other subtypes (Figures 1E, S1E, and S1F). These observations suggested that the PML/RARα targets exhibited M3 subtype specific expression patterns and could help identify more M3 patients. Moreover, we found that certain samples from other subtypes were clustered together with patients of M3 subtype, implying that these patients have more similar expression patterns as M3 subtype, although they do not have the PML/RARα fusion event.

Together, these results suggested that the expression of PML/RARα targets was likely to be perturbed in multiple AML subtypes and the expression patterns of PML/RARα targets can greatly help identify patients similar as M3 subtype.

### M3-LS model accurately predicts M3 subtype in AML

**Figure 2:**
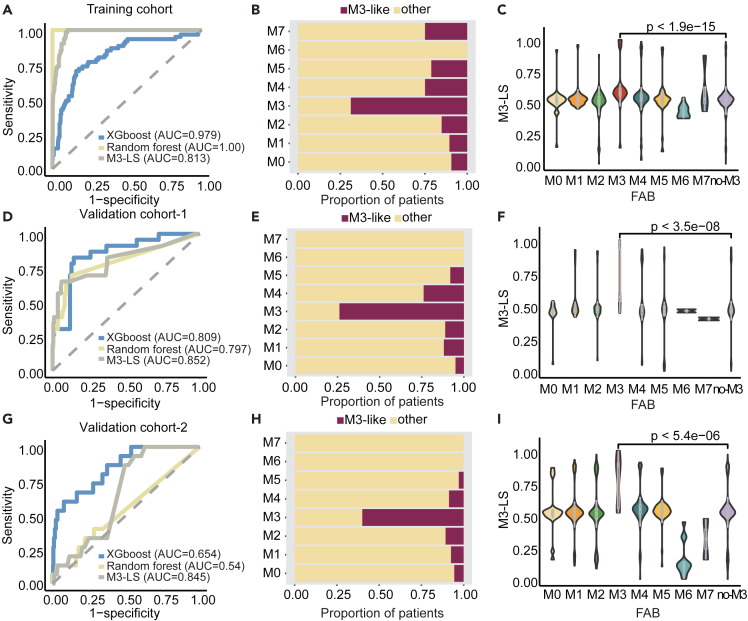
M3-LS model accurately predicts M3 subtype in AML (A) Random forest, XGBoost, and M3-LS model were used to predict M3 samples, and Receiver operating characteristic curve (ROC) analysis was used to evaluate the prediction model. (B) The proportion of M3-like samples predicted by the optimized model in each subtype, amaranth represents the proportion of samples predicted to be M3-like subtype, and yellow represents the proportion of samples not predicted to be M3-like subtype. (C) Model scores were compared for each AML subtype. Boxes and violin plots showing median, 25th and 75th percentiles. Purple box and violin plots represent model scores for all AML samples except M3 subtype. Wilcoxon Rank-Sum test was used for statistical calculation. Validation cohort-1 and 2, (D and G**)** Random forest, XGBoost machine learning models and M3-like scoring index were used to predict M3 samples. (E and H) The proportion of M3-like samples predicted by the optimized model in each subtype. (F and I) Model scores were compared for each AML subtype.

We next hypothesized that if PML/RARα-activated target genes were more likely to be upregulated in a patient, whereas repressed target genes were likely to be downregulated, the patient was more similar to M3 subtype. A computational model, M3-LS, based on the expression pattern of PML/RARα targets was developed to predict patients of M3 subtype. We next applied M3-LS model to the training AML cohort (Table 1), and found that the M3-LS can accurately distinguish patients in M3 subtype from other subtypes with an AUC 0.813 (Figure 2A). Next, we also trained random forest and XGboost models using the M3-LS as features in the training cohort, and the AUCs of two classifiers reached 1.00 and 0.979 (Figure 2A), respectively. The sensitivity reached 0.841 when the normalized M3-LS was 0.560. Based on this cutoff, we predicted M3 patients, and 73% of patients of M3 subtype were successfully predicted (Figure 2B). In addition, the normalized M3-LS in patients of M3 subtype were the highest compared to other subtypes (Figure 2C, p < 1.9e-15, Wilcoxon’s rank-sum test).

Moreover, the M3-LS model was found to successfully distinguish patients of M3 subtype from other subtypes in two independent AML cohorts assayed by different platforms. In the first validation cohort, the AUC scores respectively reached 0.852, 0.797, and 0.809 for three classifiers (Figure 2D). Similarly, approximately 75% of patients of M3 subtype were successfully predicted (Figure 2E), and their scores were also significantly higher than those in other subtypes (Figure 2F, p < 3.5e-08, Wilcoxon’s rank-sum test). Our model was also validated in the second cohort (Figures 2G–2I). Thus, these results indicated that M3-LS model integrating the expression patterns with regulation information could accurately predict M3 subtype in AML from the view of transcriptome. That is in addition to the genomic event of PML/RARα fusion, perturbed expression patterns of its target genes can also reflect the molecular signature of M3 subtype.

### Performance of M3-LS model is improved by integrating ATO/ATRA response genes

**Figure 3:**
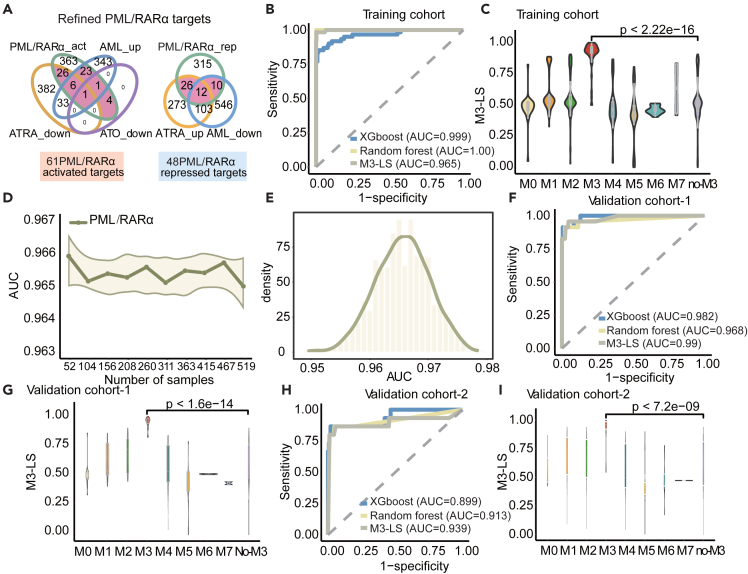
Performance of M3-LS model was improved by integrating ATO/ATRA response genes (A) Venn plot of model optimization, including leading edge genes (LEGs) of ATO, differential genes robust rank aggregation results of ATRA, PML/RARα target genes, and differential expression genes of M3 and healthy controls in the training cohort. (B) Random forest, XGBoost machine learning model and optimized M3-LS index were used to predict M3 samples, and ROC analysis was used to evaluate the prediction model in the training cohort. (C) Comparison of the scores of the optimized models for each subtype. (D) The model was validated using ROC analysis. Use the model to predict several randomly selected samples, the line graph represents the size of the AUC. (E) Probability density distribution plot of AUC. (F) Random forest, XGBoost machine learning model and optimized M3-LS index were used to predict M3 samples, and ROC analysis was used to evaluate the prediction model in the validation cohort-1. (G) Comparison of model scores for each subtype in the validation cohort-1. (H) Random forest, XGBoost machine learning model and optimized M3-LS index were used to predict M3 samples, and ROC analysis was used to evaluate the prediction model in the validation cohort-2. (I) Comparison of model scores for each subtype in the validation cohort-2. Statistics were calculated using Wilcoxon Rank-Sum test.

The combination of ATO and ATRA is a landmark treatment regimen in M3 AML.^32^^,^^33^ An increasing number of studies have also revealed that the treatment process can alter the expression of PML/RARα target genes, and subsequently perturb the downstream biological functions.^10^^,^^20^^,^^22^ We next explored to what extent the M3-LS model can be refined by integration of ATO and ATRA treatment datasets (Table 2). We first obtained drug-response genes after treated with ATO or ATRA, as well as abnormally expressed genes in M3 patients. There were 448/414 genes significantly down/up-regulated by ATRA treatment (Figure 3A). In addition, 61 genes were detected to respond to the treatment of ATO, and 671/407 genes were down-regulated/up-regulated in M3 AML when compared with normal samples (Figure 3A). Combined with the above gene set of PML/RARα targets, we obtained 109 refined target genes, including 61 activated and 48 repressed genes (Figure 3A).

Then, the M3-LS model was re-trained based on these refined PML/RARα target genes, we found that M3 patients in the training cohort can be distinguished from other subtypes with higher accuracy (AUC = 0.965, Figure 3B). In particular, the AUCs of the refined random forest and XGboost classifiers respectively reached 1.00 and 0.999 (Figure 3B). Approximately 86.89% of M3 patients were successfully predicted, and their scores were significantly higher than other subtypes (Figure 3C, p < 2.22e-16, Wilcoxon’s rank-sum test).

The robustness of our M3-LS model was evaluated from three aspects. First, we randomly used 10%–100% patients to train the model and evaluate the effects of sample size. It was found that our model can reach high AUCs in different numbers of patients (Figure 3D), even in a small number of patients. Second, considering the relatively large size of non-M3 patients compared with M3 patients, we next randomly selected the same number of non-M3 patients as M3 to eliminate the imbalance effects, and these processes were repeated 1000 times. Our model can also obtain higher AUC values ranging from 0.95 to 0.98 (Figure 3E). Finally, the great improvements of our models were discovered in other two validation cohorts (Figures 3F–3I) and the AUC values reached up to 0.99 and 0.939 respectively (Figures 3F and 3H). Similarly, M3 patients exhibited significantly higher normalized M3-LS than other patients (Figures 3G and 3I). All these results supported that integration PML/RARα targets with ATO/ATRA response genes could further refine our model, and reveal that M3 subtype could be distinguished from AML at the transcriptome level.

### M3-LS model identifies additional patients like M3 subtype

**Figure 4:**
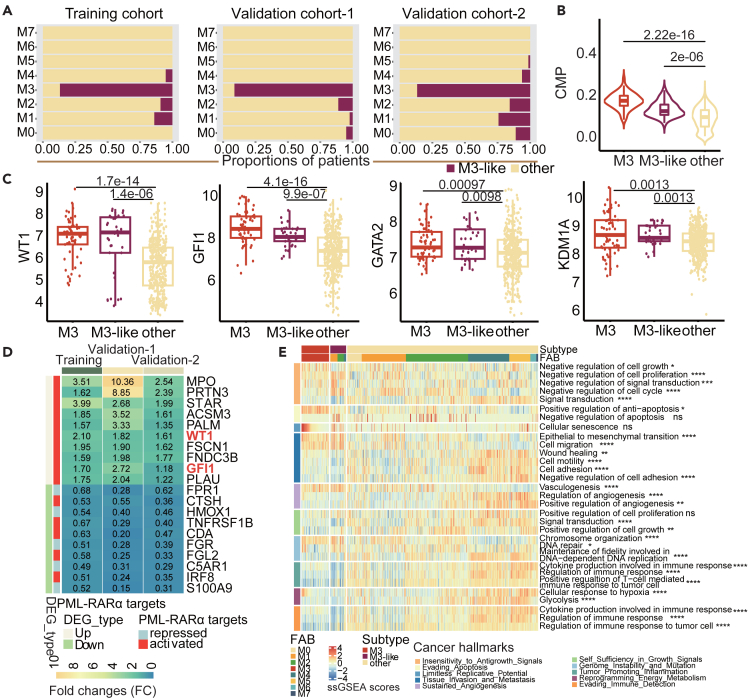
M3-LS model identifies additional patients like M3 subtype (A) The proportion of M3-like samples predicted by the optimized model in each subtype in the training and validation cohorts, respectively. Amaranth represents the proportion of samples predicted to be M3-like subtype, and yellow represents the proportion of samples not predicted to be M3-like subtype. (B) Violin plot of the proportion of common myeloid progenitor (CMP) of each subtype identified in the training cohort. Boxes plots show median, 25th and 75th percentiles of CMP for each subtype. p values are calculated using Kruskal-Wallis Test. (C) The expression levels of WT1, GFI1, GATA2, and KDM1A of each subtype were compared AML cases with predicted as M3-like versus M3 subtype and other samples in the training cohort. p value was estimated using Kruskal-Wallis Test. (D) The differential expression of PML/RARα target genes in M3-like and other samples in the training and validation cohorts. The heatmap shows the fold change (FC) values of differential genes in M3-like samples relative to other samples, and the genes in red font are characteristic genes of M3 subtype. (E) Cancer Hallmark pathway enrichment of M3 subtype, M3-like subtype and other samples. The heatmap shows the results of single sample gene set enrichment analysis (ssGSEA) of each subtype sample in each Cancer Hallmark pathway (Statistical significance was assessed by Wilcoxon Rank-Sum test, ∗p < 0.05, ∗∗p < 0.01, ∗∗∗p < 0.001, ∗∗∗∗p < 0.0001). Data are represented as mean.

Based on the observations that M3-LS model can accurately predict M3 patients, we next predicted M3-like AML patients in three cohorts. That is if a patient of non-M3 subtype was predicted to be positive one, the patient was considered to form an additional subtype named M3-like. In total, there were M3-like patients from 7.6% non-M3 subtypes in the training cohort, accounting for 14.04% of M1 subtype, 9.21% of M2, and 5.2% of M4 (Figure 4A). In addition, 3.61% and 12.37% AML patients in two validation cohorts were also predicted as M3-like subtype, respectively (Figure 4A).

We next sought to understand the relevance of these defined M3-like patients to the functional, biological and clinical properties of M3 subtype. First, it was well known that AML is a malignant disease of myeloid progenitor cells.^34^ We thus applied the xCell^35^ method to estimate the proportion of myeloid progenitor cells in AML patients. As a result, patients in both M3 and M3-like subtypes exhibited a much higher common myeloid progenitor (CMP) scores than the other subtypes (Figure 4B, p < 2.2e-16, Wilcoxon’s rank sum tests). Moreover, several marker genes of M3 subtype exhibited significantly higher expressions in M3-like patients, such as *WT1*, *GFI1*, *GATA2* and *KDM1A* (Figure 4C). For example, *WT1*, as an activated target gene of PML/RARα, was not only over-expressed in AML as described above, but also was repressed by both ATO and ATRA. *WT1* has been found to be an important regulator of normal and malignant hematopoiesis, which is usually inactivated in APL patients and results in the complete loss of WT1’s inhibitory function on APL tumor cells.^36^ We also observed higher expression of *GATA2* in M3 and M3-like subtypes, which has been demonstrated as a prognosis factor in AML.^21^ The combination of *KDM1A* inhibitor and ATRA can promote the induction and differentiation of leukemia cells by ATRA.^37^ We found that the expression levels of *KDM1A* were significantly increased in M3 and M3-like subtypes. Significantly high expression of these genes was also discovered in the validation sets (Figures S3A–S3J).

To explore the related molecular function of M3-like subtype, differentially expressed genes were first identified, and Figure 4D showed the 10 most significantly differentially expressed genes in the training and validation cohorts, respectively. Among them, *WT1* and *GFI1* are PML/RARα target genes. Notably, the target genes activated by PML/RARα were all up-regulated in M3-like subtype, while the target genes inhibited by PML/RARα were mostly down-regulated in M3-like subtype (Figure 4D). These findings suggested that the PML/RARα targets expression patterns of M3-like samples were highly similar to those of M3 subtype. Both carcinogenesis and immune related biological functions were further explored in AML patients by single sample gene set enrichment analysis (ssGSEA). The cancer hallmark-associated pathways were obtained from the literature^38^ and the MSigDB database.^39^ Globally, patients in M3 and M3-like subtypes exhibited similar pathway activities across cancer hallmarks (Figures 4E and S3K). The patients in M3-like subtype were found to be enriched in six particular functions, including ‘Negative regulation of cell proliferation’, ‘Negative regulation of cell cycle’, ‘Epithelial to mesenchymal transition’, ‘Cell migration’, ‘Vasculogenesis’ and ‘Chromosome organization’, which were related to ‘Insensitivity to Antigrowth Signals’, ‘Tissue Invasion and Metastasis’, ‘Sustained Angiogenesis’ and ‘Genome Instability and Mutation’ cancer hallmarks. For the cancer hallmark-related pathways, the patients in M3 and M3-like subtypes were mostly enriched in pathways related to signal regulation, including WNT beta-catenin signaling, Notch signaling, Estrogen response early, TGF beta-signaling and Estrogen response late (Figure S3K). Thus, these findings revealed that multiple properties of M3-like patients were much more similar to M3 ones.

### M3-like patients with strong GMP and distinct genomic features

**Figure 5:**
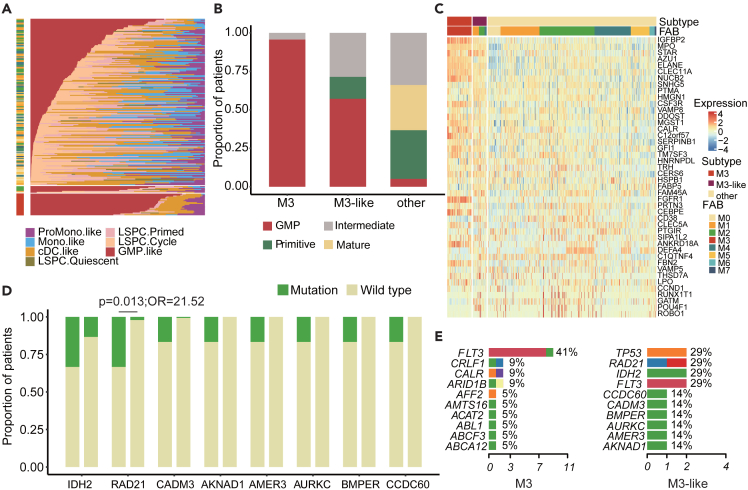
M3-like patients with strong GMP and distinct genomic features (A) Relative abundance of each leukemic cell type per patient. Each bar represents a patient, and the distribution of colors on each bar represents the distribution of the leukemia cell populations within their leukemic hierarchy. (B) Hierarchical classification of leukemia cells for each subtype in the training cohort. (C) In the training cohort, the expression of the GMP -like marker genes of M3 subtype, M3-like subtype and other samples. (D) Mutation frequency of some genes in M3-like subtype (left) and other sample (right). Statistical significance was assessed by Fisher’s test. (E) Top 10 genes with mutation frequency in M3 subtype and M3-like subtype.

A recent study has demonstrated that the cellular hierarchy composition constitutes a novel framework for understanding disease biology and advancing precision medicine in AML.^40^ We thus evaluated the cellular compositions of the AML patients. In total, the abundance of seven leukemic cell types was estimated by a deconvolution approach, three of which were leukemia stem and progenitor cells (LSPCs), namely Quiescent LSPCS, Primed LSPCS, and Cycling LSPCS. The other four leukemia cell types were GMP-like blasts, ProMono-like blasts, Mono-like blasts and cDC-like blasts, which were classified by a recent study.^41^ Based on the leukemia hierarchy composition, we revealed four distinct subtypes: Primitive (shallow hierarchy, LSPC-enriched), Mature (steep hierarchy, enriched for mature Mono-like and cDC-like blasts), GMP (dominated by GMP-like blasts) and Intermediate (balanced distribution). We found that patients in M3 and M3-like subtypes exhibited a higher proportion of GMP-like cells (Figure 5A). Moreover, the majority of patients of M3 and M3-like subtypes were classified as GMP subtypes (Figure 5B). By analyzing the expression of GMP-like marker genes, we found that these genes were more likely to be highly expressed in both M3 and M3-like patients (Figure 5C). For instance, the expression level of *IGFBP2* is high in leukemia (Figure S4A). Inhibition of endogenous *IGFBP2* expression in human leukemia cells leads to increased apoptosis, decreased migration, and decreased activation of *AKT* and other signaling molecules.^42^ *MPO* is generally considered to be the definitive marker of myeloblasts. Targeting *MPO* expression or enzyme activity sensitizes AML cells to cytarabine therapy by triggering oxidative damage and persistent oxidative stress, especially in AML cells with high *MPO* expression^43^ (Figure S4B). We also observed higher expression of *CLEC11A* in M3 and M3-like subtypes (Figure S3C). TCGA data showed that high expression of *CLEC11A* was associated with a good prognosis^44^ (Figure S4C).

To better understand the genomic features of M3-like subtype, we analyzed the somatic mutations in the patients of validation cohort-1 (Figure S4D; Table S1). Generally, the mutation burden of M3 and M3-like subpopulation was relatively higher than other ones (Figure S4E). On the one hand, several genes exhibited higher mutation frequency in M3 patients (Figure S4F), such as *FLT3* and *ARID1B*. On the other hand, distinct genomic features were found in M3-like patients, such as *IDH2*, *RAD21* and *CADM3* (Figure 5D). *IDH2* mutation was not detected in M3-like subtype, and it has been shown that the vulnerability of *IDH2* mutation in AML leads to sensitivity to APL-like targeted combination therapy.^33^ *RAD21* was mutated in M3-like patients, which were more likely to be mutated in M3-like patients (Figure 5D, p = 0.013 and OR = 21.52). *RAD21* is a complete subunit of the eukaryotic cohesive complex that regulates chromosome separation and DNA damage response.^45^ *RAD21* mutation sensitized patients to treatment with the *BCL2* inhibitor ABT-199, and reducing *RAD21* levels sensitized AML cells to *BCL2* inhibition.^46^ In detail, we found that *FLT3*, *CRLF1* and *CALR* exhibited higher mutation frequency in M3 patients (Figure 5E), and *TP53*, *RAD21*, *IDH2*, and *FLT3* exhibited higher frequency in M3-like patients (Figure 5E). Furthermore, we found specific *CCDC60*, *BMPER*, *AMER3*, *AURKC*, and *AKNAD1* mutations only in M3-like subtype (Figure 5E). For example, *CCDC60* is a member of the coiled-coil domain containing family, which takes part in the occurrence and development of many types of cancer.^47^^,^^48^^,^^49^ *AURKC* is a member of the aurora subfamily of serine/threonine protein kinases and may play a role in mitosis. It has been shown that single nucleotide polymorphisms in *AURKC* were associated with cancer risk in both glioblastoma and gastric cancer.^50^^,^^51^ These specific mutations could be used to define M3-like subtype. These results suggested that M3-like and M3 patients were highly similar in terms of GMP-like cells, and the abnormal genomic features were distinct.

### M3-like patients with low immune activity and better clinical survival

**Figure 6:**
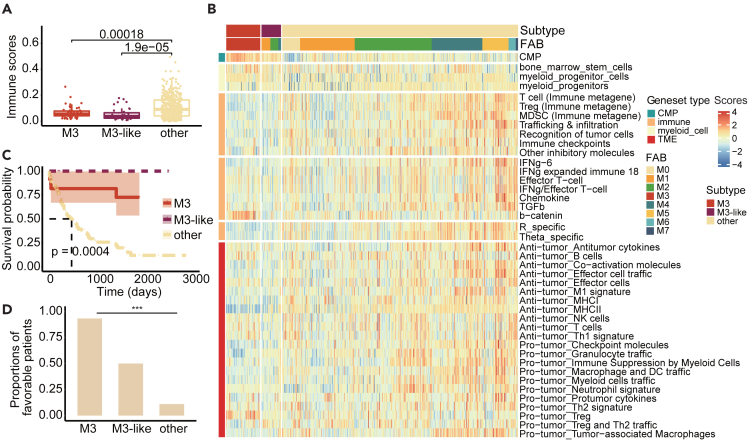
M3-like patients with low immune activity and better clinical survival (A) Immune scores for each subtype were calculated using Xcell. Boxplots show median, 25th and 75th percentiles of immunity scores for each subtype. p values are calculated using Kruskal-Wallis Test. (B) In the training cohort, enrichment of various immune gene cohorts and myeloid gene cohorts for M3 subtype, M3-like subtype and other samples. The heatmap shows the results of ssGSEA of each subtype sample in each gene cohort. (C) Kaplan-Meier survival analysis of AML cases predicted as M3-like versus M3 subtype and other samples in the validation cohort-1. p-values were estimated using the log rank test. (D) Percentage of favorable patients for each subtype in the validation cohort-1 (Statistical significance was assessed by Fisher’s test, ∗p < 0.05, ∗∗p < 0.01, ∗∗∗p < 0.001, ∗∗∗∗p < 0.0001). Data are represented as mean.

Immunotherapy modulating the tumor microenvironment (TME) has a promising effect on AML,^52^ but the therapy effects depend on the TME of patients. We next sought to determine whether the TMEs of M3-like patients were distinct from other subtypes. Immune scores were estimated in the training cohort by xCell,^35^ and the relatively low immune scores of patients in M3 and M3-like subtypes were discovered, which were significant (Figure 6A, P < 3e−07 by Kruskal-Wallis Test). A similar situation was found in both validation cohorts (Figures S5A and S5B), suggesting that M3-like patients had lower immune activity than M3 patients. Moreover, we explored the expressions of LM22 immunotherapy gene sets in the training cohort, and also found that these genes exhibited significantly lower expressions in patients of M3 and M3-like subtypes (Figure S5C).

Moreover, we used ssGSEA to estimate the abundance of cell types and the activities of particular gene sets. Interestingly, the proportions of myeloid cells in M3 and M3-like patients were higher (Figure 6B), and the b-catenin signaling pathway related to immunotherapy was also enriched in most M3 patients. In human metastatic melanoma samples, there is a correlation between the activation of the b-catenin signaling pathway in tumors and the absence of T cell gene expression signature, which leads to the mechanism of immunotherapy resistance.^53^ In contrast, M3 and M3-like patients were less enriched for other immune-related gene sets (Figure 6B). Moreover, in validation cohort-2, M3 and M3-like patients also had lower enrichment of immune-related gene sets, except for myeloid cell-related gene sets (Figure S5D).

Finally, the clinical correlations were explored, we found that patients with different subtypes exhibited significantly distinct survival outcomes, in line with the observed associations with Cancer and Acute Leukemia Group B (CALGB) cytogenetics risk category, and patients in M3 and M3-like subtypes had better clinical survival (Figure 6C, p = 0.0004, log rank test). Moreover, there were higher proportions of patients with favorable outcomes in M3 and M3-like subtypes (Figure 6D, p < 2.2e-16, Fisher’s exact test). So, M3-like patients were characterized by low infiltration of immune cells and better clinical survival outcome.

## Discussion

In this study, we developed a novel computational model to discover M3-like subtype of AML based on expression features of PML/RARα targets. Our analysis found that the expression of PML/RARα targets was frequently perturbed across AMLs and helped identify M3 subtype. Previous studies have shown that some AML patients with *IDH2* mutations respond well to ATRA and ATO combination therapy, although they may not have the PML/RARα fusion protein.^33^ Therefore, we hypothesized that non-M3 patients with high expression of PML/RARα up-regulated target genes and low expression of down-regulated target genes were likely to be M3 subtype. Our computational model can not only distinguish patients of M3 subtype, but also can further predict a set of samples with similar expression patterns to M3 subtype.

Notably, several results suggest that these M3-like patients are more consistent with M3 subtype, such as the expression patterns of several important marker genes of M3 subtype, the proportion of myeloid progenitor cells, as well as deconvolution of AML constituent cell populations. Furthermore, we found that M3-like patients exhibit some molecular features that differ from other non-M3-like patients, including genomic mutations and molecular immune signatures. Benefiting from the high efficiency of ATRA and ATO combined therapy, the survival prognosis of M3 patients is generally superior to that of other subtypes.^9^ Interestingly, we found that the clinical prognosis of M3-like samples was similar to that of M3 samples and significantly better than that of other samples. Moreover, an unexpected finding of our study was that both M3 subtype and M3-like subtype tend to have low immune characteristics, which is also a possibility that they are not suitable for immunotherapy, further indicating that they might be suitable for targeted therapy.

The most widely accepted treatment regimen of M3 subtype is the classic targeted combination therapy of ATO/ATRA, and their cure rate is up to 95%.^6^^,^^7^ Therefore, expanding this treatment plan to more types of AML can enable more leukemia patients to be treated effectively. Our model performance was improved by further requiring the PML/RARα targets to respond to ATRA/ATO or to be differentially expressed in M3 subtype. In addition, we also found that treatment did not significantly affect the expression of PML/RARα targets and the efficacy of the model. The Jaccard-coefficient of differentially expressed genes between treatment and diagnostic groups and PML/RARα targets was very low, only 0.0188. The AUC of the reconstructed model only based on diagnostic samples was 0.96. However, there are still some challenges in the optimization process. ATRA-treated cell lines collected by us were those of M3 subtype with higher consistency, while the ATO-treated cell lines were derived from multiple human tissues and were heterogeneous. Hence, we used different methods to extract ATRA and ATO target genes. If data on ATO/ATRA medication were consistent in the background of M3 subtype, our model could be further improved. Additionally, we tried to find M3-like cells in existing cell lines for testing the efficacy of ATRA and ATO. However, we found no cell lines with high M3-LS except for NB4 (M3 type) (Table S3). In future studies, we will try to construct M3-like primary cells to validate the model.

A large number of targeted therapies for AML are currently being developed, and great progress has been made in targeted therapies for M3 patients. We believed that the initiative of identifying patients similar to M3 subtype in our study may help to find patients who would benefit from ATO/ATRA treatment and deepen our understanding of AML pathogenesis.

### Limitations of the study

There are still several challenges in the optimization process. Our collections of ATRA-treated cell lines were those of M3 subtype with higher consistency, while the ATO-treated cell lines were derived from multiple human tissues and were heterogeneous. Hence, we used different methods to extract ATRA and ATO target genes. If data on ATO/ATRA medication were consistent in the background of M3 subtype, our model could be further improved.

## STAR★Methods

### Key resources table

**Table:** 

REAGENT or RESOURCE	SOURCE	IDENTIFIER
**Biological samples**		

See Table 1	This study	N/A
See Table 2	This study	N/A

**Software and algorithms**		

R version 4.0.2 or higher	Open source	https://www.r-project.org/
GSEA (version 1.28.0 in R)	Open source	N/A
randomForest (version 4.6.12 in R)	Open source	N/A
xgboost (version 1.7.6.1 in R)	Open source	N/A
survival (version 3.1.12 in R)	Open source	N/A
ggplot2 (version 3.3.5 in R)	Open source	N/A

### Resource availability

#### Lead contact

The relevant experimental methods, and related data of this study can be obtained by contacting Juan Xu (xujuanbiocc@ems.hrbmu.edu.cn).

#### Materials availability

The study did not generate new materials.

#### Data and code availability

• This paper analyzes existing, publicly available data. These accession numbers for the datasets are listed in the Tables 1 and 2.

**Table 1:** Characteristics of AML patients

	Training cohort (n = 519)	Validation cohort-1 (n = 217)	Validation cohort-2 (n = 492)
Sample type	Bone marrow	Bone marrow	Bone marrow
Platform	Microarray	RNA-seq	Microarray
Sample source	GEO(9)^a^	TCGA&cBioportal^b^	GEO(5)^c^

**French-American-British (FAB) classification(n)**			

M0	32	20	18
M1	114	42	107
M2	152	45	140
M3	61	23	15
M4	96	46	127
M5	47	37	70
M6	13	2	13
M7	4	2	2

**Cytogenetic(n)**			

Poor	unknown	46	unknown
Intermediate	unknown	107	unknown
Favorable	unknown	45	unknown

a GSE10358, GSE61804, GSE68833, GSE12662, GSE12417, GSE37642.

b http://v15.proteinatlas.org/about/download.

c GSE83449, GSE9476, GSE12417, GSE34860, GSE37642.

**Table 2:** Cell lines treated with ATO or ATRA

Cell line	Accession	Platform	Sample number
**ATO treatment**			

ASPC1	GSE124069	GPL570	1 control, 3 case
PANC1	GSE124069	GPL570	1 control, 3 case
BXPC3	GSE124069	GPL570	1 control, 4 case
MIApaca	GSE124069	GPL570	1 control, 4 case
U266	GSE14519	GPL570	2 control, 3 case
MM.1s	GSE14519	GPL570	2 control, 3 case
KMS11	GSE14519	GPL570	2 control, 3 case
8226/S	GSE14519	GPL570	2 control, 3 case
BEAS-2B	GSE33520	GPL6480	4 control, 3 case
Jurkat	GSE46909	GPL16311	4 control, 4 case
HepG2	GSE48441	GPL201	3 control, 15 case
HepG2	GSE6907	GPL201	3 control, 3 case
MEC-1	GSE78207	GPL16987	3 control, 3 case
HepG2	GSE8865	GPL201	3 control, 3 case
hESC	GSE94521	GPL570	5 control, 5 case
K562	GSE104813	GPL10332	1 control, 1 case
KU-812	GSE104813	GPL10332	1 control, 1 case
MEG-A2	GSE104813	GPL10332	1 control, 1 case

**ATRA treatment**			

HL-60	GSE34726	GPL10558	6 control, 6 case
NB4	GSE23702	GPL6244	3 control, 6 case
HL-60	GSE5007	GPL96	3 control, 3 case
• All original code is available in this paper’s supplemental information.
• Any additional information required to reanalyze the data reported in this paper is available from the lead contact upon request.

### Method details

#### Transcriptome data of AML patients

We collected transcriptome data of AML patients and normal samples from Gene Expression Omnibus (GEO), The Cancer Genome Atlas (TCGA), cBioportal^54^ and ArrayExpress.^55^ In total, 1228 AML patients and 271 healthy bone marrow (BM) samples covering 15 datasets were collected (Table 1). As the transcriptomes were assayed by three different platforms, we next divided them into three independent cohorts, including 519 AML and 122 normal samples by HG-U133 2.0 microarray (training cohort), 217 samples by RNA-seq (validation cohort-1), and 492 samples by HG-U133A microarray (validation cohort-2).

Microarray data were preprocessed based on RMA normalization by the affy R package as previously published literature.^56^^,^^57^ The RNA-seq datasets were downloaded from TCGA data portal (https://tcga-data.nci.nih.gov/tcga/) and cBioportal for Cancer Genomics (https://www.cbioportal.org/datasets). Gene expressions were quantified as fragments per kilobase of transcript per million (FPKM).

We collected transcriptome data of 24 AML cell lines from Cancer Cell Line Encyclopedia (CCLE).

#### Genomic mutations and clinical information of AML patients

We collected both genomic mutation data and clinical data for AML samples in the validation cohort-1.^58^ In addition, clinical information of other AML patients was collected from their corresponding metadata files or literatures in GEO or ArrayExpress.

#### PML/RARα target genes and ATRA/ATO response-related gene signatures

The PML/RARα target gene signatures were obtained from a previous study.^5^ To identify ATRA/ATO response-related genes, we obtained the expression profiles of 21 cell lines treated with ATO or ATRA and corresponding control datasets from GEO (Table 2). For each dataset, fold change (FC) of each gene as a ratio of averages from samples treated with ATRA/ATO and control was first calculated by R package limma.^59^ To obtain the ATRA response genes, genes were further ranked according to their FC for each ATRA dataset. Lastly, the Robust Rank Aggregation (RRA)^60^ method was used to integrate these ranked results of three datasets. ATRA response-related genes were defined as significantly differentially expressed genes in the RRA analysis (FDR <0.2 and fold change > 1.5). To obtain the ATO response genes, for each dataset, gene set enrichment analysis (GSEA)^61^ was performed to obtain the leading edge genes (LEGs) by using ranked all genes based on their FC values. LEGs were considered as potential candidate genes responded to ATO. Lastly, ATO response-related genes were defined as those candidate genes which were identified in at least six datasets.

#### Functional gene sets

Myeloid cell-related gene sets were obtained from published literature,^62^ including myeloid cell genes, myeloid progenitor cell genes, bone marrow cell genes, and neutrophil genes (Table S4). In addition, we collected multiple immune-related gene sets, including gene sets associated with immune infiltration,^63^ immune-related gene sets,^64^ immunotherapy response-related gene sets,^65^ and tumor microenvironment gene sets^66^ (Table S4).

#### A computational model for identifying M3 and M3-like AML patients

To identify M3 and M3-like AML patients based on transcriptome, we proposed a two-step computational model. Briefly, an enrichment-based scoring index, M3-Like Score (M3-LS), was proposed to assess the expression similarity of PML/RARα target genes in non-M3 AML patients as M3 ones. Second, a classifier was developed based on the M3-LS index to distinguish both M3 and M3-like patients.

For each AML patient k, all genes were ranked based on FCs which were calculated by comparing expression levels to the average expression levels in normal samples. We next used GSEA to assess whether the PML/RARα activated or repressed target genes were observed at the top or bottom of the ranked gene list in each patient, respectively. The gene set activated by PML/RARα or gene set repressed by PML/RARα was used as a gene set to perform GSEA. The enrichment level for patient k was measured by the enrichment scores, ${\text{ES}}_{\text{down}}\left(k\right)$ or ${\text{ES}}_{\text{up}}\left(k\right)$. ${\text{ES}}_{\text{down}}$ evaluates the enrichment level of PML/RARα directly activated targets in the top of the ranked gene list, and ${\text{ES}}_{\text{up}}$ evaluates the enrichment level of PML/RARα directly repressed targets in the bottom of the gene list. Moreover, M3-LS was calculated by integrating these two scores as follows:

$$M3-\text{LS}\left(k\right)={\text{ES}}_{\text{down}}\left(k\right)-{\text{ES}}_{\text{up}}\left(k\right)$$

The M3-LS for all patients were further normalized in the range from 0 to 1:

$$M3-{\text{LS}}^{\prime }\left(k\right)=\frac{M3-\text{LS}\left(k\right)-\min\left(M3-\text{LS}\left(i\right)\right)}{\max\left(M3-\text{LS}\left(i\right)\right)-\min\left(M3-\text{LS}\left(i\right)\right)},i=1,2,3,\ldots ,n$$

where n was the number of patients. If the normalized M3-LS is closer to 1, the expression patterns of PML/RARα targets in this sample are more similar to M3 patients.

Finally, we trained the classifiers based on normalized M3-LS as features to identify M3-like AML patients. The code used in our model is available in Data S1 and GitHub (https://github.com/Ljning666/A-machine-learning-model-identifies-M3-like-subtype-in-AML-based-on-PML-RAR-targets).

We explored to what extent the M3-LS model can be refined by integration of ATO and ATRA treatment datasets. Refined PML/RARα target genes were constructed by integrating the above gene set of PML/RARα targets, abnormally expressed genes in M3 patients and ATRA/ATO response-related gene signatures. The M3-LS model was re-trained based on these refined PML/RARα target genes. The refined PML/RARα targets were used as gene sets to perform GSEA.

#### Evaluation of the M3-LS computational model

The classifiers were evaluated based on five-fold cross-validation. The prediction efficiency was assessed in terms of sensitivity, specificity and the overall prediction accuracy. The receiver operating characteristic (ROC) curve is a robust approach for classifier evaluation and is drawn by plotting sensitivity against the false-positive rate, which equals 1-specificity. The area under the ROC curve (AUC) was used as a reliable measure of classifier performance, which was calculated by the R package pROC version 1.18.0.

Moreover, the robust of our proposed model was assessed from two aspects. First, the effect of sample size was measured by randomly gradually increasing samples from 10% to all ones with an increase of 10%, which were used to train our model. Second, to evaluate the effects of imbalance sizes between M3 subtype and other ones, the model was constructed by randomly selecting the same number of non-M3 patients as of M3 samples. Then the AUC values of the model were calculated. These processes were repeated 1,000 times.

Here, XGboost and random forests classifiers were used to verify the effectiveness of the M3-LS. All samples in the training cohort (Table 1) were classified into M3 and non-M3 samples according to the sample subtype label. The M3-LS of each sample was calculated based on the expression of PML/RARα targets. We trained random forest and XGboost using the M3-LS as features to predict sample label (M3 samples or non-M3 samples) in the training cohort, respectively. Validation cohort-1 and validation cohort-2 were used as validation datasets.

#### Immune activity analysis

The ssGSEA method was used to evaluate the activity of each immune gene set in each sample by using the R package GSVA,^67^ and the activities of gene sets across samples were visualized as a heatmap. In addition, gene expressions in the immune gene sets were also visualized using the R package ComplexHeatmap.^68^

#### Survival analysis

Overall survival was measured from the date of diagnosis to the date of last follow-up or death. All survival analyses were performed using the survival package (version 3.31), with survival curves visualized using the survminer package (version 0.4.9).

### Quantification and statistical analysis

ClusterProfiler^69^ package in R software was used for functional enrichment analysis, and Gene Ontology (GO) biological processes with a significant level (FDR < 0.05) were employed. Hypergeometric test was performed using R function phyper. Both Wilcoxon rank-sum test and Kruskal-Wallis test were used to calculate statistical differences between control vehicle and treatment group. All statistical analyses were performed with R software (Version 4.1.0).

## Supplemental information

Document S1. Figures S1–S6 and Data S1

Table S1. A table with mutation frequency of genes in the validation cohort-1, related to Figure 5E Top 10 genes with mutation frequency in M3 subtype and M3-like subtype

Table S2. The mutation and karyotype information of the samples in the validation cohort-1, related to Figure 5D Mutation frequency of some genes in M3-like subtype (left) and other sample (right)

Table S3. M3-LS of 24 AML cell lines from the CCLE database, related to Method details

Table S4. The gene list of gene sets obtained from references, related to Figure 6B In the training cohort, enrichment of various immune gene cohorts and myeloid gene cohorts for M3 subtype, M3-like subtype and other samples

## References

[1] Papaemmanuil E., Gerstung M., Bullinger L., Gaidzik V.I., Paschka P., Roberts N.D., Potter N.E., Heuser M., Thol F., Bolli N. (2016). Genomic Classification and Prognosis in Acute Myeloid Leukemia. N. Engl. J. Med..

[2] Metzeler K.H., Herold T., Rothenberg-Thurley M., Amler S., Sauerland M.C., Görlich D., Schneider S., Konstandin N.P., Dufour A., Bräundl K. (2016). Spectrum and prognostic relevance of driver gene mutations in acute myeloid leukemia. Blood.

[3] Mason E.F., Kuo F.C., Hasserjian R.P., Seegmiller A.C., Pozdnyakova O. (2018). A distinct immunophenotype identifies a subset of NPM1-mutated AML with TET2 or IDH1/2 mutations and improved outcome. Am. J. Hematol..

[4] Lin X., Qiao N., Shen Y., Fang H., Xue Q., Cui B., Chen L., Zhu H., Zhang S., Chen Y. (2021). Integration of Genomic and Transcriptomic Markers Improves the Prognosis Prediction of Acute Promyelocytic Leukemia. Clin. Cancer Res..

[5] Tan Y., Wang X., Song H., Zhang Y., Zhang R., Li S., Jin W., Chen S., Fang H., Chen Z., Wang K. (2021). A PML/RARalpha direct target atlas redefines transcriptional deregulation in acute promyelocytic leukemia. Blood.

[6] Shen Z.X., Shi Z.Z., Fang J., Gu B.W., Li J.M., Zhu Y.M., Shi J.Y., Zheng P.Z., Yan H., Liu Y.F. (2004). All-trans retinoic acid/As2O3 combination yields a high quality remission and survival in newly diagnosed acute promyelocytic leukemia. Proc. Natl. Acad. Sci. USA.

[7] Hu J., Liu Y.F., Wu C.F., Xu F., Shen Z.X., Zhu Y.M., Li J.M., Tang W., Zhao W.L., Wu W. (2009). Long-term efficacy and safety of all-trans retinoic acid/arsenic trioxide-based therapy in newly diagnosed acute promyelocytic leukemia. Proc. Natl. Acad. Sci. USA.

[8] Lo-Coco F., Avvisati G., Vignetti M., Thiede C., Orlando S.M., Iacobelli S., Ferrara F., Fazi P., Cicconi L., Di Bona E. (2013). Retinoic acid and arsenic trioxide for acute promyelocytic leukemia. N. Engl. J. Med..

[9] Dores G.M., Devesa S.S., Curtis R.E., Linet M.S., Morton L.M. (2012). Acute leukemia incidence and patient survival among children and adults in the United States, 2001-2007. Blood.

[10] Di Martino O., Niu H., Hadwiger G., Kuusanmaki H., Ferris M.A., Vu A., Beales J., Wagner C., Menéndez-Gutiérrez M.P., Ricote M. (2021). Endogenous and combination retinoids are active in myelomonocytic leukemias. Haematologica.

[11] Brown G., Hughes P. (2012). Retinoid differentiation therapy for common types of acute myeloid leukemia. Leuk. Res. Treat..

[12] Bacher U., Schnittger S., Haferlach T. (2010). Molecular genetics in acute myeloid leukemia. Curr. Opin. Oncol..

[13] Yan J., Wang K., Dong L., Liu H., Chen W., Xi W., Ding Q., Kieffer N., Caen J.P., Chen S. (2010). PML/RARalpha fusion protein transactivates the tissue factor promoter through a GAGC-containing element without direct DNA association. Proc. Natl. Acad. Sci. USA.

[14] Coltella N., Percio S., Valsecchi R., Cuttano R., Guarnerio J., Ponzoni M., Pandolfi P.P., Melillo G., Pattini L., Bernardi R. (2014). HIF factors cooperate with PML-RARalpha to promote acute promyelocytic leukemia progression and relapse. EMBO Mol. Med..

[15] Wang K., Wang P., Shi J., Zhu X., He M., Jia X., Yang X., Qiu F., Jin W., Qian M. (2010). PML/RARalpha targets promoter regions containing PU.1 consensus and RARE half sites in acute promyelocytic leukemia. Cancer Cell.

[16] Yang X.W., Wang P., Liu J.Q., Zhang H., Xi W.D., Jia X.H., Wang K.K. (2014). Coordinated regulation of the immunoproteasome subunits by PML/RARalpha and PU.1 in acute promyelocytic leukemia. Oncogene.

[17] Nasr R., Lallemand-Breitenbach V., Zhu J., Guillemin M.C., de Thé H. (2009). Therapy-induced PML/RARA proteolysis and acute promyelocytic leukemia cure. Clin. Cancer Res..

[18] Zhu J., Lallemand-Breitenbach V., de Thé H. (2001). Pathways of retinoic acid- or arsenic trioxide-induced PML/RARalpha catabolism, role of oncogene degradation in disease remission. Oncogene.

[19] Lehmann-Che J., Bally C., Letouzé E., Berthier C., Yuan H., Jollivet F., Ades L., Cassinat B., Hirsch P., Pigneux A. (2018). Dual origin of relapses in retinoic-acid resistant acute promyelocytic leukemia. Nat. Commun..

[20] Duprez E., Wagner K., Koch H., Tenen D.G. (2003). C/EBPbeta: a major PML-RARA-responsive gene in retinoic acid-induced differentiation of APL cells. EMBO J..

[21] Katerndahl C.D.S., Rogers O.R.S., Day R.B., Cai M.A., Rooney T.P., Helton N.M., Hoock M., Ramakrishnan S.M., Nonavinkere Srivatsan S., Wartman L.D. (2021). Tumor suppressor function of Gata2 in acute promyelocytic leukemia. Blood.

[22] Smitheman K.N., Severson T.M., Rajapurkar S.R., McCabe M.T., Karpinich N., Foley J., Pappalardi M.B., Hughes A., Halsey W., Thomas E. (2019). Lysine specific demethylase 1 inactivation enhances differentiation and promotes cytotoxic response when combined with all-trans retinoic acid in acute myeloid leukemia across subtypes. Haematologica.

[23] Trus M.R., Yang L., Suarez Saiz F., Bordeleau L., Jurisica I., Minden M.D. (2005). The histone deacetylase inhibitor valproic acid alters sensitivity towards all trans retinoic acid in acute myeloblastic leukemia cells. Leukemia.

[24] Tayari M.M., Santos H.G.D., Kwon D., Bradley T.J., Thomassen A., Chen C., Dinh Y., Perez A., Zelent A., Morey L. (2021). Clinical Responsiveness to All-trans Retinoic Acid Is Potentiated by LSD1 Inhibition and Associated with a Quiescent Transcriptome in Myeloid Malignancies. Clin. Cancer Res..

[25] Bullinger L., Schlenk R.F., Götz M., Botzenhardt U., Hofmann S., Russ A.C., Babiak A., Zhang L., Schneider V., Döhner K. (2013). PRAME-induced inhibition of retinoic acid receptor signaling-mediated differentiation--a possible target for ATRA response in AML without t(15;17). Clin. Cancer Res..

[26] Kelly L.M., Kutok J.L., Williams I.R., Boulton C.L., Amaral S.M., Curley D.P., Ley T.J., Gilliland D.G. (2002). PML/RARalpha and FLT3-ITD induce an APL-like disease in a mouse model. Proc. Natl. Acad. Sci. USA.

[27] Lian X., Lin Y.M., Kozono S., Herbert M.K., Li X., Yuan X., Guo J., Guo Y., Tang M., Lin J. (2018). Pin1 inhibition exerts potent activity against acute myeloid leukemia through blocking multiple cancer-driving pathways. J. Hematol. Oncol..

[28] Lange T., Hubmann M., Burkhardt R., Franke G.N., Cross M., Scholz M., Leiblein S., Al-Ali H.K., Edelmann J., Thiery J., Niederwieser D. (2011). Monitoring of WT1 expression in PB and CD34(+) donor chimerism of BM predicts early relapse in AML and MDS patients after hematopoietic cell transplantation with reduced-intensity conditioning. Leukemia.

[29] Inoue K., Sugiyama H., Ogawa H., Nakagawa M., Yamagami T., Miwa H., Kita K., Hiraoka A., Masaoka T., Nasu K. (1994). WT1 as a new prognostic factor and a new marker for the detection of minimal residual disease in acute leukemia. Blood.

[30] Lin S.Y., Hu F.F., Miao Y.R., Hu H., Lei Q., Zhang Q., Li Q., Wang H., Chen Z., Guo A.Y. (2019). Identification of STAB1 in Multiple Datasets as a Prognostic Factor for Cytogenetically Normal AML: Mechanism and Drug Indications. Mol. Ther. Nucleic Acids.

[31] Karikoski M., Marttila-Ichihara F., Elima K., Rantakari P., Hollmén M., Kelkka T., Gerke H., Huovinen V., Irjala H., Holmdahl R. (2014). Clever-1/stabilin-1 controls cancer growth and metastasis. Clin. Cancer Res..

[32] de Almeida L.Y., Pereira-Martins D.A., Weinhäuser I., Ortiz C., Cândido L.A., Lange A.P., De Abreu N.F., Mendonza S.E.S., de Deus Wagatsuma V.M., Do Nascimento M.C. (2021). The Combination of Gefitinib With ATRA and ATO Induces Myeloid Differentiation in Acute Promyelocytic Leukemia Resistant Cells. Front. Oncol..

[33] Mugoni V., Panella R., Cheloni G., Chen M., Pozdnyakova O., Stroopinsky D., Guarnerio J., Monteleone E., Lee J.D., Mendez L. (2019). Vulnerabilities in mIDH2 AML confer sensitivity to APL-like targeted combination therapy. Cell Res..

[34] De Kouchkovsky I., Abdul-Hay M. (2016). Acute myeloid leukemia: a comprehensive review and 2016 update. Blood Cancer J..

[35] Aran D., Hu Z., Butte A.J. (2017). xCell: digitally portraying the tissue cellular heterogeneity landscape. Genome Biol..

[36] Song H., Liu Y., Tan Y., Zhang Y., Jin W., Chen L., Wu S., Yan J., Li J., Chen Z. (2022). Recurrent noncoding somatic and germline WT1 variants converge to disrupt MYB binding in acute promyelocytic leukemia. Blood.

[37] Ravasio R., Ceccacci E., Nicosia L., Hosseini A., Rossi P.L., Barozzi I., Fornasari L., Zuffo R.D., Valente S., Fioravanti R. (2020). Targeting the scaffolding role of LSD1 (KDM1A) poises acute myeloid leukemia cells for retinoic acid-induced differentiation. Sci. Adv..

[38] Plaisier C.L., Pan M., Baliga N.S. (2012). A miRNA-regulatory network explains how dysregulated miRNAs perturb oncogenic processes across diverse cancers. Genome Res..

[39] Liberzon A., Birger C., Thorvaldsdóttir H., Ghandi M., Mesirov J.P., Tamayo P. (2015). The Molecular Signatures Database (MSigDB) hallmark gene set collection. Cell Syst..

[40] Zeng A.G.X., Bansal S., Jin L., Mitchell A., Chen W.C., Abbas H.A., Chan-Seng-Yue M., Voisin V., van Galen P., Tierens A. (2022). A cellular hierarchy framework for understanding heterogeneity and predicting drug response in acute myeloid leukemia. Nat. Med..

[41] van Galen P., Hovestadt V., Wadsworth Ii M.H., Hughes T.K., Griffin G.K., Battaglia S., Verga J.A., Stephansky J., Pastika T.J., Lombardi Story J. (2019). Single-Cell RNA-Seq Reveals AML Hierarchies Relevant to Disease Progression and Immunity. Cell.

[42] Chen X., Zheng J., Zou Y., Song C., Hu X., Zhang C.C. (2013). IGF binding protein 2 is a cell-autonomous factor supporting survival and migration of acute leukemia cells. J. Hematol. Oncol..

[43] Hosseini M., Rezvani H.R., Aroua N., Bosc C., Farge T., Saland E., Guyonnet-Dupérat V., Zaghdoudi S., Jarrou L., Larrue C. (2019). Targeting Myeloperoxidase Disrupts Mitochondrial Redox Balance and Overcomes Cytarabine Resistance in Human Acute Myeloid Leukemia. Cancer Res..

[44] Yin C., Zhang J., Guan W., Dou L., Liu Y., Shen M., Jia X., Xu L., Wu R., Li Y. (2021). High Expression of CLEC11A Predicts Favorable Prognosis in Acute Myeloid Leukemia. Front. Oncol..

[45] Xu H., Balakrishnan K., Malaterre J., Beasley M., Yan Y., Essers J., Appeldoorn E., Tomaszewski J.M., Vazquez M., Verschoor S. (2010). Rad21-cohesin haploinsufficiency impedes DNA repair and enhances gastrointestinal radiosensitivity in mice. PLoS One.

[46] Bisaillon R., Moison C., Thiollier C., Krosl J., Bordeleau M.E., Lehnertz B., Lavallée V.P., MacRae T., Mayotte N., Labelle C. (2020). Genetic characterization of ABT-199 sensitivity in human AML. Leukemia.

[47] Liu Z., Chen S., Jia W., Qian Y., Yang X., Zhang M., Fang T., Liu H. (2023). Comprehensive analysis reveals CCDC60 as a potential biomarker correlated with prognosis and immune infiltration of head and neck squamous cell carcinoma. Front. Oncol..

[48] Li C.F., Wu W.R., Chan T.C., Wang Y.H., Chen L.R., Wu W.J., Yeh B.W., Liang S.S., Shiue Y.L. (2017). Transmembrane and Coiled-Coil Domain 1 Impairs the AKT Signaling Pathway in Urinary Bladder Urothelial Carcinoma: A Characterization of a Tumor Suppressor. Clin. Cancer Res..

[49] Geng W., Liang W., Fan Y., Ye Z., Zhang L. (2018). Overexpression of CCDC34 in colorectal cancer and its involvement in tumor growth, apoptosis and invasion. Mol. Med. Rep..

[50] Mesic A., Rogar M., Hudler P., Bilalovic N., Eminovic I., Komel R. (2021). Genetic variations in AURORA cell cycle kinases are associated with glioblastoma multiforme. Sci. Rep..

[51] Mesic A., Rogar M., Hudler P., Juvan R., Komel R. (2016). Association of the AURKA and AURKC gene polymorphisms with an increased risk of gastric cancer. IUBMB Life.

[52] Huerga-Domínguez S., Villar S., Prósper F., Alfonso-Piérola A. (2022). Updates on the Management of Acute Myeloid Leukemia. Cancers.

[53] Spranger S., Bao R., Gajewski T.F. (2015). Melanoma-intrinsic beta-catenin signalling prevents anti-tumour immunity. Nature.

[54] Gao J., Aksoy B.A., Dogrusoz U., Dresdner G., Gross B., Sumer S.O., Sun Y., Jacobsen A., Sinha R., Larsson E. (2013). Integrative analysis of complex cancer genomics and clinical profiles using the cBioPortal. Sci. Signal..

[55] Athar A., Füllgrabe A., George N., Iqbal H., Huerta L., Ali A., Snow C., Fonseca N.A., Petryszak R., Papatheodorou I. (2019). ArrayExpress update - from bulk to single-cell expression data. Nucleic Acids Res..

[56] Warnat-Herresthal S., Perrakis K., Taschler B., Becker M., Baßler K., Beyer M., Günther P., Schulte-Schrepping J., Seep L., Klee K. (2020). Scalable Prediction of Acute Myeloid Leukemia Using High-Dimensional Machine Learning and Blood Transcriptomics. iScience.

[57] Gautier L., Cope L., Bolstad B.M., Irizarry R.A. (2004). affy--analysis of Affymetrix GeneChip data at the probe level. Bioinformatics.

[58] Tyner J.W., Tognon C.E., Bottomly D., Wilmot B., Kurtz S.E., Savage S.L., Long N., Schultz A.R., Traer E., Abel M. (2018). Functional genomic landscape of acute myeloid leukaemia. Nature.

[59] Ritchie M.E., Phipson B., Wu D., Hu Y., Law C.W., Shi W., Smyth G.K. (2015). limma powers differential expression analyses for RNA-sequencing and microarray studies. Nucleic Acids Res..

[60] Kolde R., Laur S., Adler P., Vilo J. (2012). Robust rank aggregation for gene list integration and meta-analysis. Bioinformatics.

[61] Subramanian A., Tamayo P., Mootha V.K., Mukherjee S., Ebert B.L., Gillette M.A., Paulovich A., Pomeroy S.L., Golub T.R., Lander E.S., Mesirov J.P. (2005). Gene set enrichment analysis: a knowledge-based approach for interpreting genome-wide expression profiles. Proc. Natl. Acad. Sci. USA.

[62] Wang K., Patkar S., Lee J.S., Gertz E.M., Robinson W., Schischlik F., Crawford D.R., Schäffer A.A., Ruppin E. (2022). Deconvolving Clinically Relevant Cellular Immune Cross-talk from Bulk Gene Expression Using CODEFACS and LIRICS Stratifies Patients with Melanoma to Anti-PD-1 Therapy. Cancer Discov..

[63] Desbois M., Udyavar A.R., Ryner L., Kozlowski C., Guan Y., Dürrbaum M., Lu S., Fortin J.P., Koeppen H., Ziai J. (2020). Integrated digital pathology and transcriptome analysis identifies molecular mediators of T-cell exclusion in ovarian cancer. Nat. Commun..

[64] Kobayashi Y., Kushihara Y., Saito N., Yamaguchi S., Kakimi K. (2020). A novel scoring method based on RNA-Seq immunograms describing individual cancer-immunity interactions. Cancer Sci..

[65] Newell F., Pires da Silva I., Johansson P.A., Menzies A.M., Wilmott J.S., Addala V., Carlino M.S., Rizos H., Nones K., Edwards J.J. (2022). Multiomic profiling of checkpoint inhibitor-treated melanoma: Identifying predictors of response and resistance, and markers of biological discordance. Cancer Cell.

[66] Bagaev A., Kotlov N., Nomie K., Svekolkin V., Gafurov A., Isaeva O., Osokin N., Kozlov I., Frenkel F., Gancharova O. (2021). Conserved pan-cancer microenvironment subtypes predict response to immunotherapy. Cancer Cell.

[67] Hänzelmann S., Castelo R., Guinney J. (2013). GSVA: gene set variation analysis for microarray and RNA-seq data. BMC Bioinf..

[68] Gu Z., Hübschmann D. (2022). Make Interactive Complex Heatmaps in R. Bioinformatics.

[69] Wu T., Hu E., Xu S., Chen M., Guo P., Dai Z., Feng T., Zhou L., Tang W., Zhan L. (2021). clusterProfiler 4.0: A universal enrichment tool for interpreting omics data. Innovation.

