# Supplementary material for: A machine learning model identifies M3-like subtype in AML based on PML/RARα targets
Source: iScience. 2024 Jan 18;27(2):108947. doi: 10.1016/j.isci.2024.108947 (PMC10844831; doi:10.1016/j.isci.2024.108947)
Supplement: Document S1. Figures S1–S6 and Data S1 [file mmc1.pdf]

iScience, Volume *27*

Supplemental information

A machine learning model identifies M3-like subtype in AML based on PML/RARα targets

Tingting Shao, Jianing Li, Minghai Su, Changbo Yang, Yingying Ma, Chongwen Lv, Wei Wang, Yunjin Xie, Gang Xu, Ce Shi, Xinying Zhou, Huitao Fan, Yongsheng Li, and Juan Xu

Supplemental Materials

Figure S1: Supporting data that PML/RARα targets are perturbed across AMLs and help identify M3 subtype, related to Figure 1.

Figure S2: Supporting data that WT1 mutation has no significant effects on the expression of PML/RARα targets, related to Figure 1. C). Top: PML/RARα effects on transcriptional activities of the directly activated gene WT1.

Figure S3: Supporting data that M3-LS model identifies additional patients like M3 subtype, related to Figure 4.

Figure S4: Supporting data that M3-like patients with strong GMP and distinct genomic features, related to Figure 5.

Figure S5: Supporting data that M3-like patients with low immune activity and better clinical survival, related to Figure 6.

Figure S6: Supporting data that treatment did not affect the efficacy of the model, related to Method details.

Data S1: The code of computational model for identifying M3 and M3-like AML patients, related to Method details.

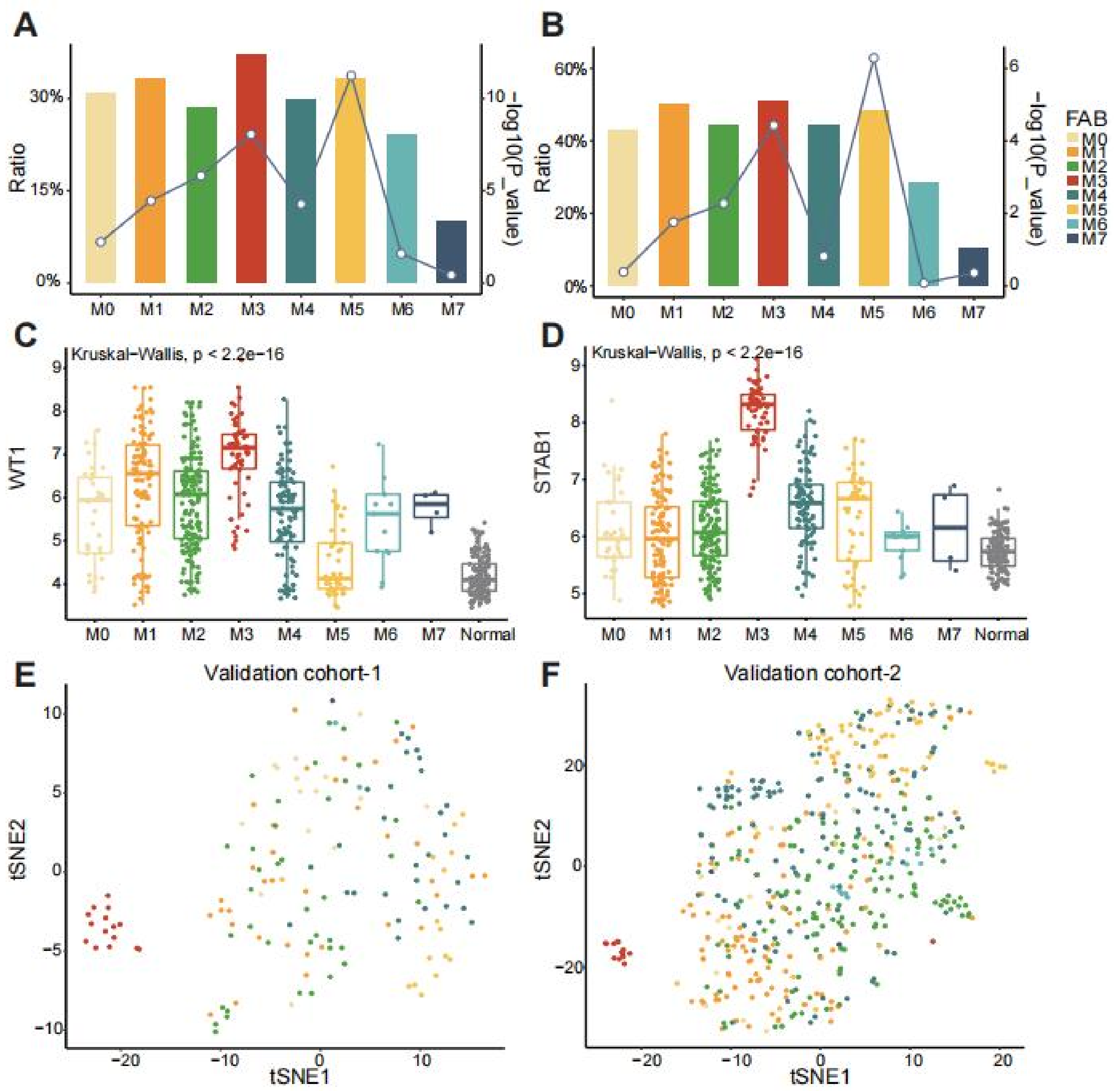

Figure S1. Supporting data that PML/RARα targets are perturbed across AMLs and help identify M3 subtype. (**A**) Enrichment of PML/RARα target genes and differential genes between AML patient and healthy control samples (FDR < 0.05, FC > 1.25). The height of the bar graph is the proportion of differentially expressed genes in the targets, and the line chart is the -log10(P value) of the hypergeometric test between the differential genes and the target genes. (**B**) Enrichment of PML/RARα target genes and the differential genes between AML patients and healthy control samples (FDR < 0.05, FC > 1.125). (**C-D**) In the training cohort, WT1 and STAB1 expression levels were compared for each AML subtype. Box and violin plots showing median, 25th and 75th percentiles. Grey box and violin plots represent WT1 or STAB1 expression levels in all AML samples except M3 subtype. Statistics were calculated using the Kruskal-Wallis test. (**E-F**) t-SNE analysis of PML/RARα target genes transcriptomic data for AML samples in the validation cohorts. Each dot represents a sample visualized in a two-dimensional projection by t-SNE. Samples of each subtype are displayed using a different color.

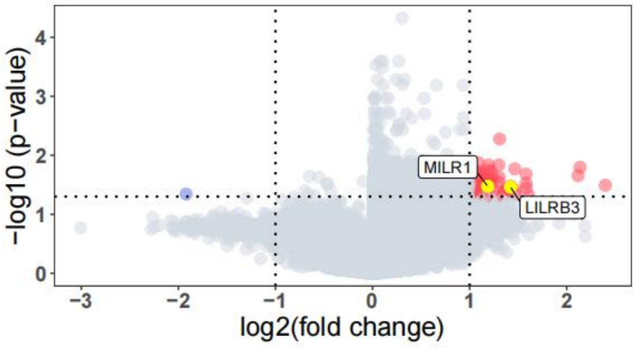

Figure S2. Supporting data that WT1 mutation has no significant effects on the expression of PML/RARα targets. Samples in the validation cohort-1 were grouped according to the mutation status of WT1 and differential expression analysis was performed between the two groups. (FDR < 0.05, FC > 2). A total of 58 differentially expressed genes were obtained. Only two PML/RARα targets(MILR1 LILRB3) were differentially expressed.

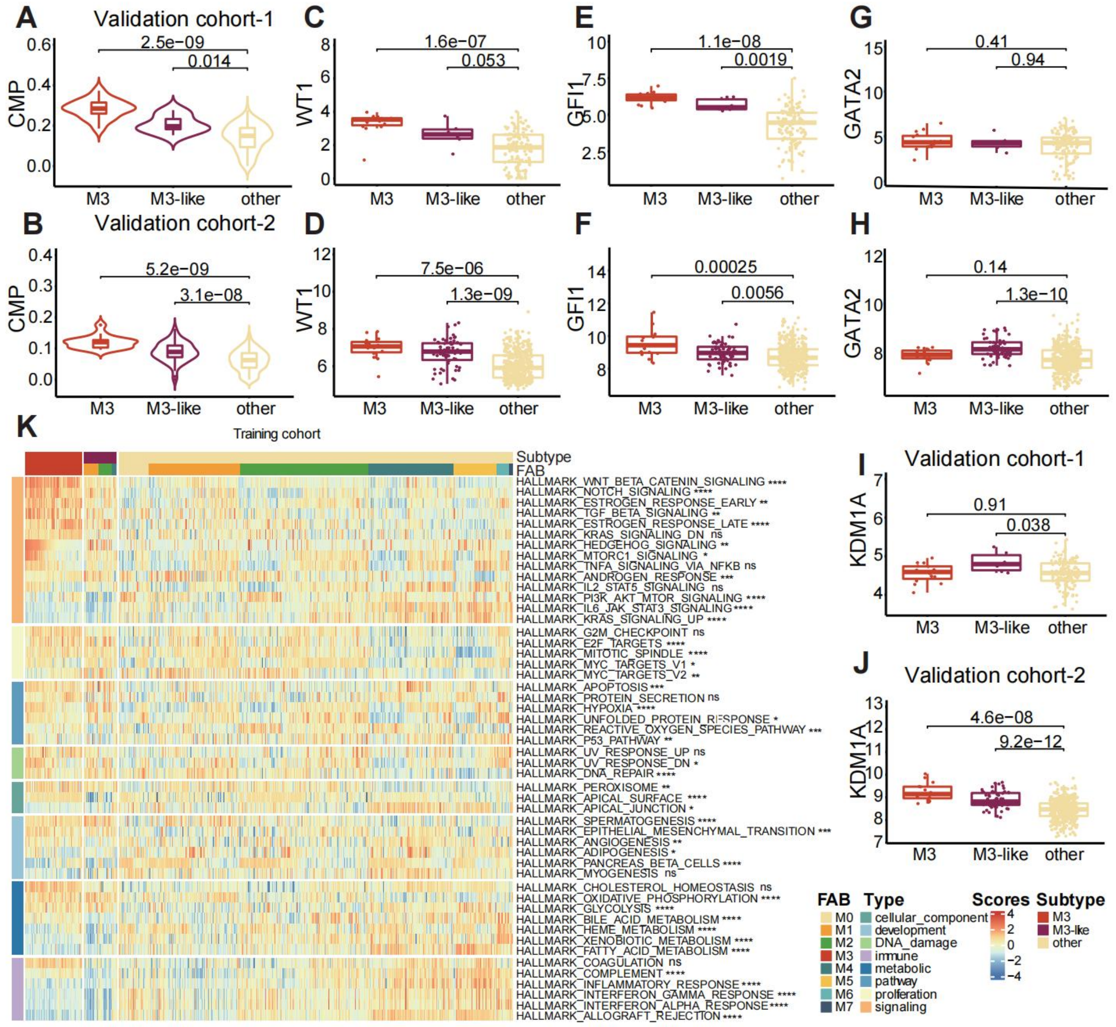

Figure S3. Supporting data that M3-LS model identifies additional patients like M3 subtype. In the validation cohorts, (**A-B**) The proportion of common myeloid progenitor (CMP) of each subtype. Box and violin plots showing median, 25th and 75th percentiles of CMP for each subtype. (**C-J**) Box plot of WT1, GFI1, GATA2 and KDM1A gene expression in M3, M3-like subtype and other samples. (**K**) Hallmark pathway enrichment of M3 subtype, M3-like subtype and other samples in the training cohort. The heatmap shows the results of ssGSEA of each subtype samples in each Hallmark pathway (Statistical significance was assessed by Kruskal-Wallis test, *p < 0.05, **p < 0.01, ***p < 0.001, ****p < 0.0001).

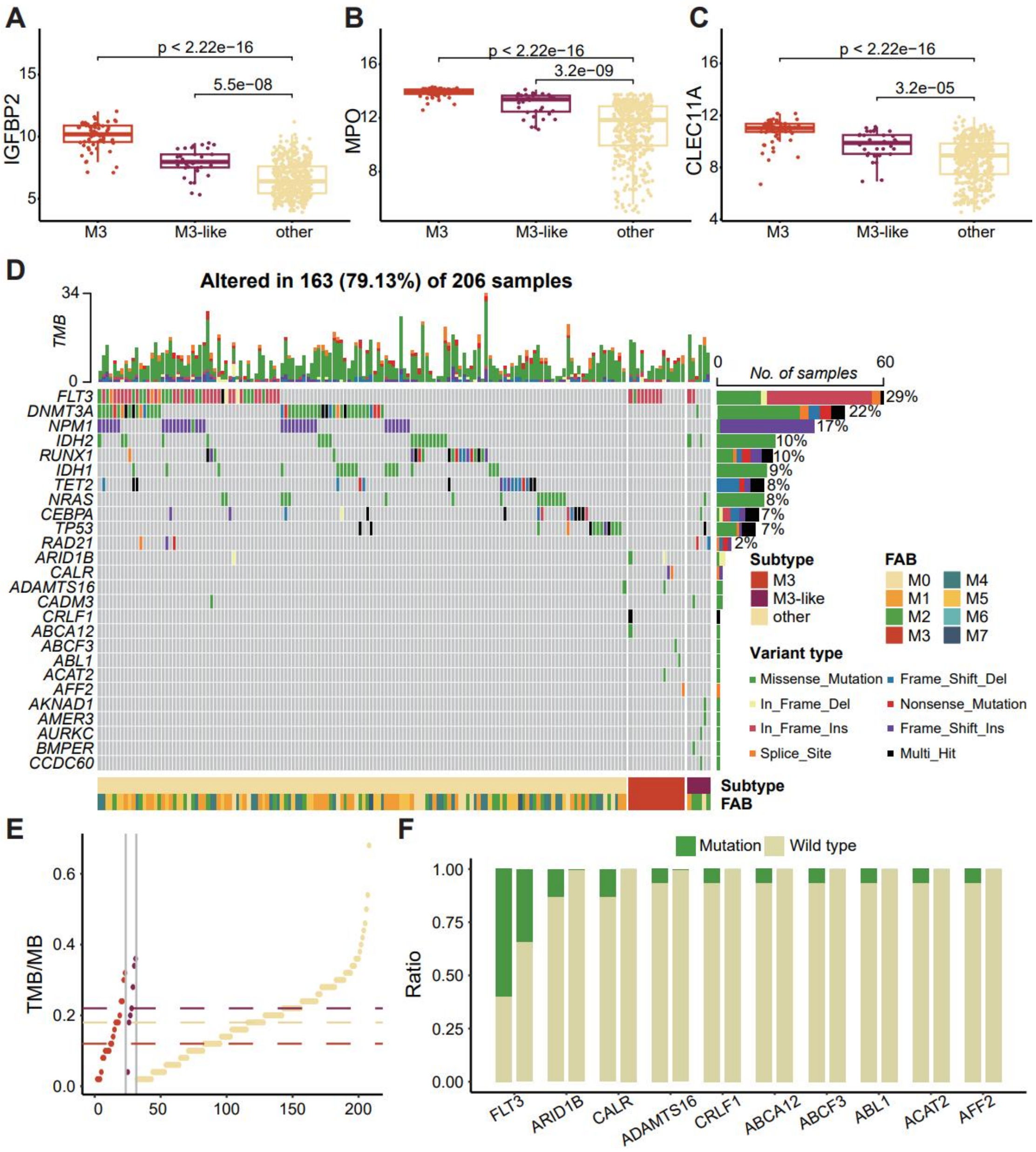

Figure S4. Supporting data that M3-like patients with strong GMP and distinct genomic features. (**A-C**) The box plot shows the expression of GMP-like marker gene IGFBP2, MPO and CLEC11A in M3, M3-like subtype and other samples. (**D**) Genes mutated in M3 subtype, M3-like subtype, and other samples are shown, and the different types of mutations are color-coded. Genes are arranged in descending order of mutation frequency. The upper bar graph shows the number of mutated genes per sample, and bars on the right plot indicate the proportion of mutations in each gene. (**E**) Tumor mutational burden (TMB) of M3 subtype, M3-like subtype, and other samples. (**F**) Mutation frequency of some genes in the M3 (left) and other (right) subtype.

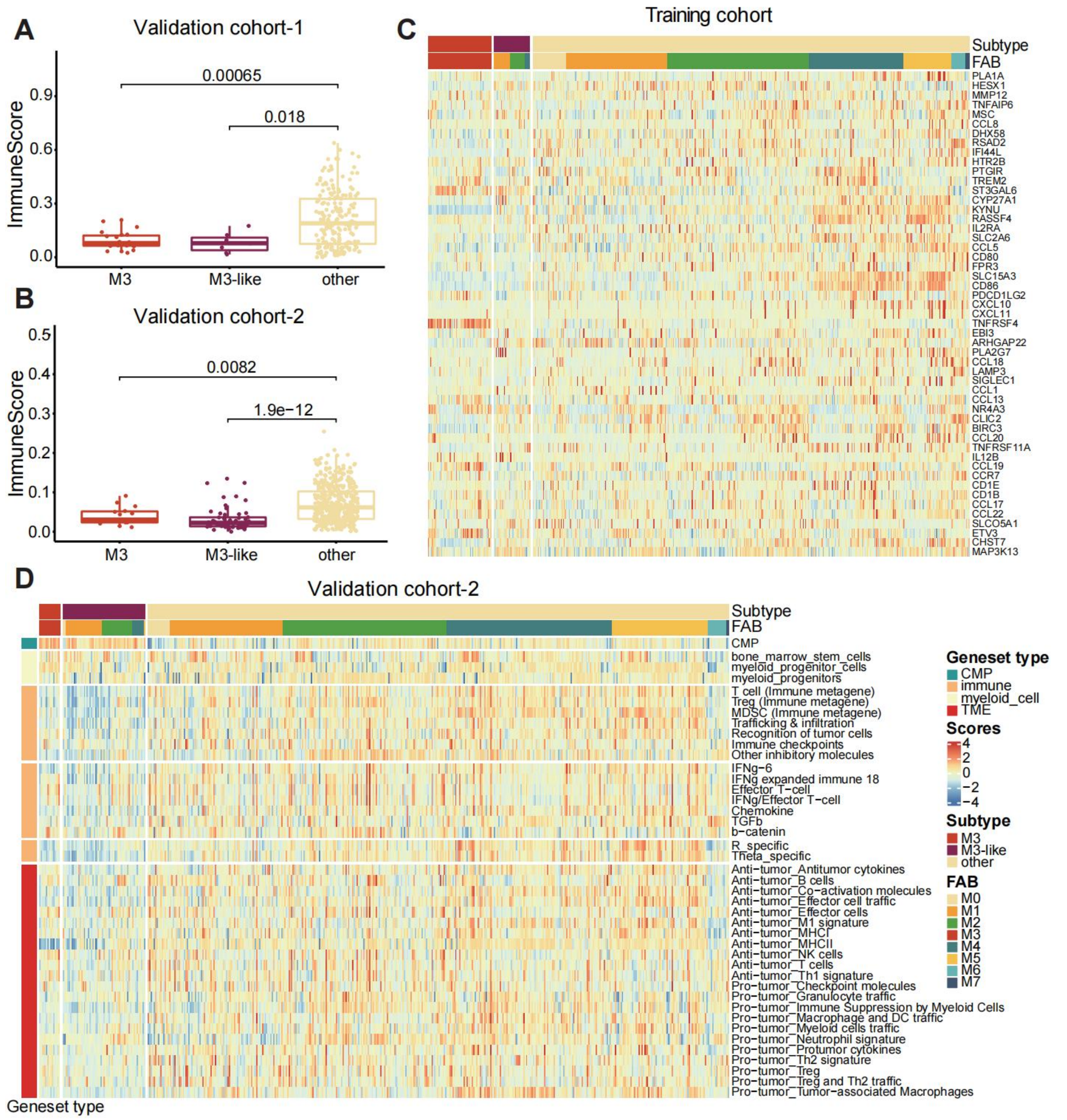

Figure S5. Supporting data that M3-like patients with low immune activity and better clinical survival. (**A-B**) Immune scores for each subtype were calculated using Xcell in the validation cohorts. Box plots show median, 25th and 75th percentiles of immunity scores for each subtype. P values are calculated using Kruskal-Wallis Test. (**C**) In the training cohort, the expression of the LM22 immunotherapy set of M3 subtype, M3-like subtype and other samples. (**D**) Enrichment of various immune gene sets and myeloid gene sets for M3 subtype, M3-like subtype and other samples in the validation cohort-2. The heatmap shows the results of single sample gene set enrichment analysis (ssGSEA) of each subtype sample in each gene set.

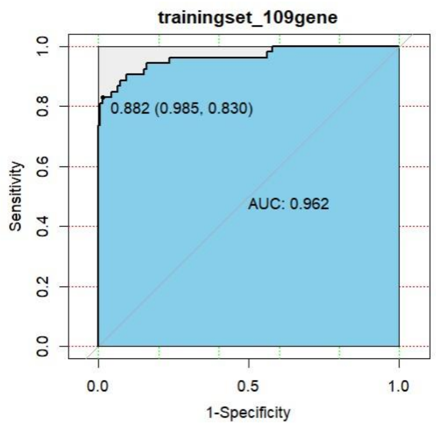

Figure S6. Supporting data that treatment did not affect the efficacy of the model. The model was validated using ROC analysis. Use the model to predict 384 diagnostic samples.

Data S1: The code of computational model for identifying M3 and M3-like AML patients.

```
library(ggplot2)
library(ggpubr)
library(fgsea)
library(pROC)
##pml_rara_geneset
target_down<-read.table("E:\\Mirror\\Classifier\\data\\PML_RARA_target\\down.txt",stringsAsFactors = F)
target_up<-read.table("E:\\Mirror\\Classifier\\data\\PML_RARA_target\\up.txt",stringsAsFactors = F)
list_down<-list(target_down=target_down$V1)
list_up<-list(target_up=target_up$V1)
AML_Sample_info<-read.delim("E:\\Mirror\\Classifier\\data\\AML_Sample_info_sur.txt",stringsAsFactors = F)
case_data1_2<-AML_Sample_info[which(AML_Sample_info$Dataset!=3&AML_Sample_info$GSE!="own"),]
case_data1_2<-case_data1_2[which(case_data1_2$Tissue=="BM"&case_data1_2$FAB!="unknown"),]
control_data1_2<-AML_Sample_info[which(AML_Sample_info$Dataset!=3&AML_Sample_info$GSE!="own"),]
control_data1_2<-control_data1_2[which(control_data1_2$Tissue=="BM"&control_data1_2$Disease=="healthy"),]
GSE122511_Dataset_2<-read.delim("E:\\Mirror\\Classifier\\data\\GSE122511_Dataset_2.txt",check.names = F,stringsAsFactors = F)
###training cohort model##########################################################
case_data2<-GSE122511_Dataset_2[,case_data1_2$GSM[which(case_data1_2$Dataset==2)]]
control_data2<-GSE122511_Dataset_2[,control_data1_2$GSM[which(control_data1_2$Dataset==2)]]
control<-control_data2
case<-case_data2
control<-2^control
case<-2^case
control<-apply(control,1,mean)
data2_logFC<-apply(case,2,function(x){return(log2(x/control))})
for(i in 1:1){
  ref<-list()
  ref[[1]]<-list_down[[i]]
  ref[[2]]<-list_up[[i]]
  names(ref)<-c("down","up")
  res2 <- apply(data2_logFC, 2, function(x){
    names(x) <- rownames(data2_logFC)
    x <-x[order(x,decreasing = T)]
    fgsea_res<-fgseaMultilevel(pathways= ref,
                                         stats=x,
```

```
                                          minSize = 1,maxSize = 2000,nproc    =    0,
                                          gseaParam    =    1,
                                          BPPARAM    =    NULL
      )
      fgsea_res2 <- c(fgsea_res$ES[1],fgsea_res$ES[2],fgsea_res$pval[1],fgsea_res$pval[2])
      return(fgsea_res2)
   }
   )
   res2<-t(res2)
   colnames(res2)<-c("down","up","down_pval","up_pval")
   res2<-as.data.frame(res2)
   res2$score<-res2$down-res2$up
   res2$score_norm<-unlist(lapply(res2$score,function(x){
      return((x-min(res2$score))/(max(res2$score)-min(res2$score)))
   }))
   res2$FAB<-AML_Sample_info$FAB[match(rownames(res2),AML_Sample_info$GSM)]
write.table(res2,"E:\\Mirror\\Classifier\\data\\PML_RARA_target\\GSEA_score\\data2_score.txt",
quote = F,sep = "\t")
###AUC
data2_score<-read.delim("E:\\Mirror\\Classifier\\data\\PML_RARA_target\\GSEA_score\\data2_
score.txt",stringsAsFactors = F)
   AUC_data<-data2_score[,6:7]
   AUC_data$FAB_type[which(AUC_data$FAB!="M3")]<-0
   AUC_data$FAB_type[which(AUC_data$FAB=="M3")]<-1
   colnames(AUC_data)<-c("score","FAB","FAB_type")
   p_AUC<-roc(AUC_data$FAB_type,AUC_data$score)
   pdf("E:\\Mirror\\Classifier\\pic\\gene787score\\data2_ROC.pdf",height = 6,width = 6)
   plot(p_AUC,print.auc=T,auc.polygon=T,
          grid=c(0.2,0.2),
          grid.col=c("green","red"),
          max.auc.polygon=T,
          legacy.axes = TRUE,
          auc.polygon.col="skyblue",
          print.thres=T,
          xlim=c(1,0),
          xlab = "1-Specificity", ylab = "Sensitivity",
          main="data2_787gene")
   dev.off()
}
data2_score$type[which(data2_score[,6]<0.560)]<-"other"
data2_score$type[which(data2_score[,6]>=0.560)]<-"M3_like"
data<-as.data.frame(table(data2_score$FAB,data2_score$type))
p<-ggplot(data,aes(Var1,Freq,fill=Var2))+
   geom_bar(stat="identity",position="fill")+
```

```
    ylab("Ratio") +
    xlab("Type")+
    theme(panel.grid=element_blank())+
    scale_fill_manual(values = c("M3_like"="#E31A1C","other"="#1F78B4"),name="Var2")+
    coord_flip()+
    ggtitle("data2_787gene")
pdf("E:\\Mirror\\Classifier\\pic\\gene787score\\data2_787gene.pdf",width=8,height=6)
print(p)
dev.off()
```
